# Role of Formyl Peptide Receptors and β-Arrestin-1 in suPAR Signal Transduction in Mouse Podocytes: Interactions with αVβ3-Integrin

**DOI:** 10.3390/cells13020172

**Published:** 2024-01-17

**Authors:** Eun Young Kim, Stuart E. Dryer

**Affiliations:** 1 Department of Biology and Biochemistry, University of Houston, Houston, TX 77204, USA; ekim8@uh.edu; 2 Department of Biomedical Sciences, Tilman J. Fertitta Family College of Medicine, University of Houston, Houston, TX 77204, USA

**Keywords:** podocytes, suPAR, formyl peptide receptors, β-arrestin, nephrotic syndrome, acute kidney injury, pattern-recognition receptor

## Abstract

The soluble urokinase plasminogen activator receptor (suPAR) has been implicated in a wide range of pathological conditions including primary nephrotic syndromes and acute kidney injuries. suPAR can trigger transduction cascades in podocytes by outside-in activation of αVβ3-integrin, but there is evidence that the functional cell surface response element is actually a complex of different types of receptors, which may also include the receptor for advanced glycation end-products (RAGE) and formyl peptide receptors (FPRs). Here we observed that ROS accumulation and Src activation could be evoked by continuous 24 h exposure to either suPAR or the FPR agonist fMLF. Responses to suPAR and fMLF were completely blocked by either the FPR antagonist WRW4 or by the αV-integrin inhibitor cilengitide. Moreover, endogenous podocyte mouse Fpr1 co-immunoprecipitates with β3-integrin, suggesting that these receptors occur as a complex on the cell surface. suPAR- and fMLF-evoked activation of Src and ROS differed in time course. Thus, robust pertussis toxin (PTX)-sensitive responses were evoked by 60 min exposures to fMLF but not to suPAR. By contrast, responses to 24 h exposures to either suPAR or fMLF were PTX-resistant and were instead abolished by knockdown of β-arrestin-1 (BAR1). FPRs, integrins, and RAGE (along with various Toll-like receptors) can all function as pattern-recognition receptors that respond to “danger signals” associated with infections and tissue injury. The fact that podocytes express such a wide array of pattern-recognition receptors suggests that the glomerular filter is designed to change its function under certain conditions, possibly to facilitate clearance of toxic macromolecules.

## 1. Introduction

The soluble urokinase plasminogen activator receptor (suPAR) is the circulating form of a plasma membrane-tethered alternatively spliced glycoprotein, known as uPAR (or CD87), that was originally defined based on its roles in regulation of the extracellular matrix, cell migration, fibrin degradation and complement fixation [1]. A large literature has described the involvement of uPAR in inflammation, immune responses, and tissue remodeling, including in the cancer microenvironment. Various forms of suPAR that range in molecular weight from 20–45 kD can be released from the cell surface through cleavage of its GPI anchor or by proteolysis [2,3]. From there, suPAR can enter extracellular compartments including blood, interstitium, and cerebral spinal fluid, and smaller suPAR variants can be detected in urine.

Circulating suPAR concentrations are elevated during a variety of grave clinical conditions, especially when inflammation is present [4,5]. Circulating suPAR is increased by immune activation, and this is closely correlated with the levels of other well-established inflammatory biomarkers such as TNF and IL-6, and with the number of circulating immune cells [5]. uPAR is expressed in multiple cell types of myeloid lineage, including monocytes, macrophages, and neutrophils [6], as well as in T-lymphocytes, endothelial cells, fibroblasts, vascular smooth muscle cells, megakaryocytes, keratinocytes, podocytes, and at least some cancer cells [7]. Any or all of those cell types are a potential source of circulating or extracellular suPAR.

Elevated circulating suPAR concentrations are strongly associated with poor patient outcomes in a remarkably broad range of clinical conditions, especially in the acute care environment, including increased risk of in-hospital-, 30-day-, and 90-day-mortality [8]. Our interest in suPAR originally stemmed from its role as one of the circulating factors that drive primary nephrotic syndromes [9]. In addition, circulating suPAR appears to exacerbate multiple forms of acute kidney injury [10,11,12]. 

It is now clear that suPAR is not simply a passive player that is coincidentally released because of other biochemical responses that drive disease processes. Rather, suPAR can exert direct effects on a wide variety of target cells through its ability to bind to cell surface receptors and thereby activate signal transduction cascades. It must be emphasized that elevated serum suPAR levels can be observed prior to adverse outcomes, for example in the critical care environment, an observation consistent with suPAR driving pathological processes, rather than simply being a marker of end-organ damage [13]. αVβ3-integrin has been the most extensively studied surface receptor for suPAR [9,14]. This integrin is expressed in a host of cells and tissues [15], including in podocytes [9,16] and in the proximal tubule [17] and it can be activated in an outside-in manner by many different ligands [18]. In podocytes, we have described a transduction pathway for suPAR that sequentially includes activation of αVβ3-integrin, activation of the small GTPase Rac1, activation of the NADPH oxidase Nox2, and generation of cytosolic reactive oxygen species (ROS) and activation of Src, which in turn induce mobilization of Ca^2+^-permeable canonical transient receptor potential-6 (TRPC6) channels to the cell surface [19,20]. 

However, there is evidence that other receptors may be involved in suPAR signal transduction. We recently described a complex between the receptor for advanced glycation end-products (RAGE) and αVβ3-integrin in podocytes, based on the observation that these proteins co-immunoprecipitate from podocyte lysates. We also observed that inhibitors of either of these receptor systems could fully antagonize signaling evoked by suPAR in mouse podocytes [21]. The idea that suPAR can exert effects on receptors other than αVβ3-integrin is not novel. Earlier studies had reported that suPAR effects on human peripheral blood monocytes [22], neutrophils [23], and vascular smooth muscle cells [24] require activation of formyl peptide receptors (FPRs), a group of G protein-coupled receptors (GPCRs), which, like RAGE, play a role in pattern recognition of various “danger signals” [25,26].

Identification and characterization of the cell surface complexes that are required for suPAR signal transduction has broader implications. In particular these types of studies suggest pharmacological strategies that allow for inhibition of suPAR signaling. Receptors on the cell surface typically have better access to inhibitors that act on intracellular targets. In this regard, G-protein coupled receptors (GPCRs) are especially attractive targets because they can typically be inhibited by small organic compounds with oral bioavailability. This is a significant advantage over agents such as monoclonal antibodies or other strategies based on immunoneutralization of suPAR. In this regard, the best characterized inhibitor of αVβ3-integrin is cilengitide [27], but this compound is a cyclic RGD peptide that cannot be used orally, and which has relatively poor pharmacokinetics. Moreover, a wide range of structural and computational tools now allow development of therapeutic agents targeting GPCRs [28,29]. In this regard, the structure of FPR2 has been solved with 2.8 Å resolution [28]. 

The aim of the present study was to examine if FPRs play a role in suPAR signal transduction in podocytes and to characterize interactions or crosstalk between FPRs and the αVβ3-integrin pathway. We now report that mouse formyl peptide receptor-1 (Fpr1) is expressed in mouse podocytes, that it co-immunoprecipitates with β3-integrin, and that suPAR and formyl-peptide signal transduction in podocytes entails a complex interplay between both receptor systems. A novel observation here is that responses to suPAR require β-arrestin-1 (BAR1), a molecule that is involved in desensitization of GPCR signaling, as well as in directing alternative signaling cascades that occur downstream and/or independent of GPCRs [30,31].

## 2. Materials and Methods

### 2.1. Cell Culture

An immortalized mouse podocyte cell line (MPC-5, passage number less than 25) was propagated and maintained at 33 °C as described previously [21,32]. Differentiation of podocytes was induced by removal of γ-interferon and temperature switch to 37 °C for 14 days. 

### 2.2. Immunoblot Analysis, Co-Immunoprecipitation, and Measurement of Cytosolic Reactive Oxygen Species

For immunoblot analyses, proteins in podocyte lysates or immunoprecipitates were separated by SDS-PAGE on 10% gels and transferred to membranes. Blots were incubated with a primary antibody overnight at 4 °C, followed by horseradish peroxidase (HRP)-conjugated secondary antibody for 1 h at room temperature. Proteins were visualized using a chemiluminescent substrate. Total Src and phosphorylated Src (Src-pY418) were obtained from ThermoFisher Scientific (Waltham, MA, USA). An antibody against β-arrestin-1 (BAR1) was obtained from Santa Cruz Biotechnology (Santa Cruz, CA, USA). Methods used for co-immunoprecipitation were described previously [32]. In one set of experiments, proteins were immunoprecipitated from lysates of untreated differentiated podocytes using an antibody against FPR1 (GeneTex, Irvine, CA, USA) or rabbit IgG. Samples of the initial lysates and the immunoprecipitates were then analyzed by immunoblot using an antibody against β3-integrin (Santa Cruz Biotechnology, sc-46655). In another set of experiments, podocyte lysates were precipitated using an antibody against BAR1 (Santa Cruz, Biotechnology, sc-53780) or IgG and the precipitates were analyzed by immunoblot using antibodies against αV-integrin (Santa Cruz Biotechnology, Santa Cruz, CA, USA, sc-9969) or β3-integrin (Santa Cruz Biotechnology, sc-46655). This same procedure was also carried out in podocytes previously treated for 24 h with 10 ng/mL of recombinant suPAR. Cytosolic abundance of reactive oxygen species (ROS) was analyzed using a fluorometric assay based on the cell permeable probe 2′,7′-dichlorodihydrofluorescein diacetate (Cell Biolabs Inc., San Diego, CA, USA).

### 2.3. SiRNA Knockdown

For transient small interfering RNA (siRNA) experiments, siRNA targeting BAR1 (sc-29742) and a non-targeted control siRNA (sc-37007) were purchased from Santa Cruz Biotechnology and transfected into podocytes using Oligofectamine™ (ThermoFisher Scientific) in serum-reduced medium according to the manufacturer’s instructions. Effectiveness of knockdown was assessed by immunoblot.

### 2.4. Recombinant Proteins and Drugs and Agonist Exposure Times

Recombinant suPAR was purchased from R&D Systems (Minneapolis, MN, USA) and was added to cell culture media at a final concentration of 10 ng/mL. FMLF and cilengitide were obtained from Tocris (Minneapolis, MN, USA). Both were used at a final concentration of 1 µM. Pertussis toxin (PTX) was obtained from Sigma-Aldrich (Saint Louis, MO, USA). WRW4 was obtained from Abcam (Boston, MA, USA). Agonists were applied for 24 h or for 60 min. Nearly all previous studies on suPAR signaling in podocytes have utilized a 24 h exposure [9,20,21]. By contrast, GPCR signaling through G proteins (as opposed to arrestins) almost always operates over a much shorter time scale often in the range of seconds to minutes. We utilized 60 min exposures to allow sufficient time for ROS accumulation in the cytosol. 

### 2.5. Statistical Methods and Quantitative Analyses

All statistical analyses were carried out using public-access computational tools (http://www.vassarstats.net) with *p* < 0.05 regarded as significant. Data were analyzed by unpaired *t*-test or by one-way ANOVA followed by Tukey’s honest significant difference post hoc test. The data in bar graphs are presented as fold changes relative to the lowest value observed in a control group and are presented as mean ± SD.

## 3. Results

All of the experiments in this study have been carried out on the MPC-5 line of immortalized podocytes that were originally developed in 1997 [33]. In the initial experiments of the present study, we examined if an FPR is also involved in suPAR signal transduction in cultured podocytes, because suPAR has been reported to activate FPR1 transduction cascades and chemotactic responses in various human cell types [22,23,24] in spite of the fact that suPAR is not a formylated protein. 

**Figure 1:**
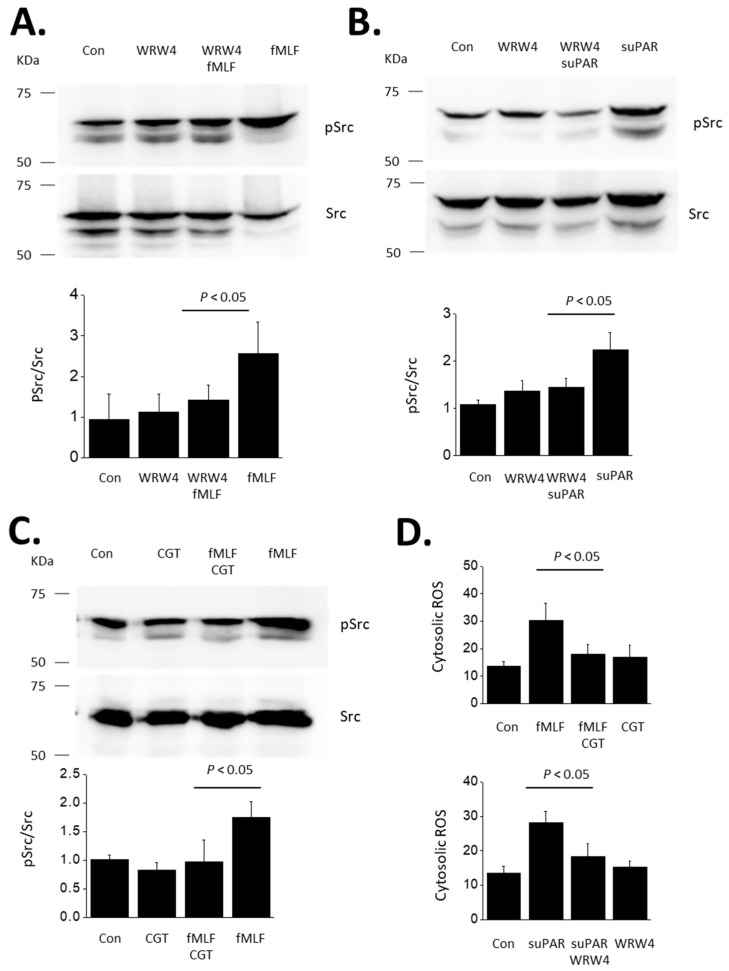
Activation of Src evoked by fMLF and suPAR in immortalized mouse podocytes. This figure shows representative immunoblots initially probed for p-Src and then stripped and probed for Src, as indicated. The bar graphs below the blots show quantitative analyses of three repetitions of this experiment. Bars indicate means normalized to the lowest value in the control groups. Error bars indicate SD. (**A**) Immunoblot analyses showing activation of Src evoked by 24 h exposure to 1 µM fMLF, and inhibition of this response by concomitant exposure to the FPR antagonist WRW4 (10 µM). (**B**) Src is also activated by 24 h exposure to 10 ng/mL of recombinant suPAR, and this response is blocked by concomitant exposure to 10 µM WRW4. (**C**) Src activation evoked by 24 h exposure to 1 µM fMLF is blocked by concomitant exposure to 1 µM cilengitide (CGT), an inhibitor of αVβ3-integrin. (**D**) CGT also blocks responses evoked by 24 h exposure to suPAR (10 ng/mL) and also inhibits responses to fMLF (1 µM). *N* = 3 for all experiments.

In our initial experiments we utilized the extensively studied FPR agonist N-formyl-Met-Leu-Phe (fMLF) to activate FPRs [34]. We observed a marked increase in the abundance of tyrosine-phosphorylated Src (p-Src) as detected by immunoblot analysis in podocytes exposed to 1 µM fMLF for 24 h compared to untreated control cells (Figure 1A). fMLF had no effect on total Src. This effect of fMLF was completely blocked in cells simultaneously exposed to 10 µM of the peptide Trp-Arg-Trp-Trp-Trp-Trp (WRW4), an antagonist of FPRs [35,36]. From these results we conclude that cultured mouse podocytes express a functional FPR capable of signaling to Src. As we have described previously, treatment with 10 ng/mL suPAR for 24 h also evoked an increase in Src phosphorylation (Figure 1B). An important observation in the present study is that this effect of suPAR was also completely blocked by WRW4, which implies a role for an FPR in suPAR-evoked Src activation. 

**Figure 2:**
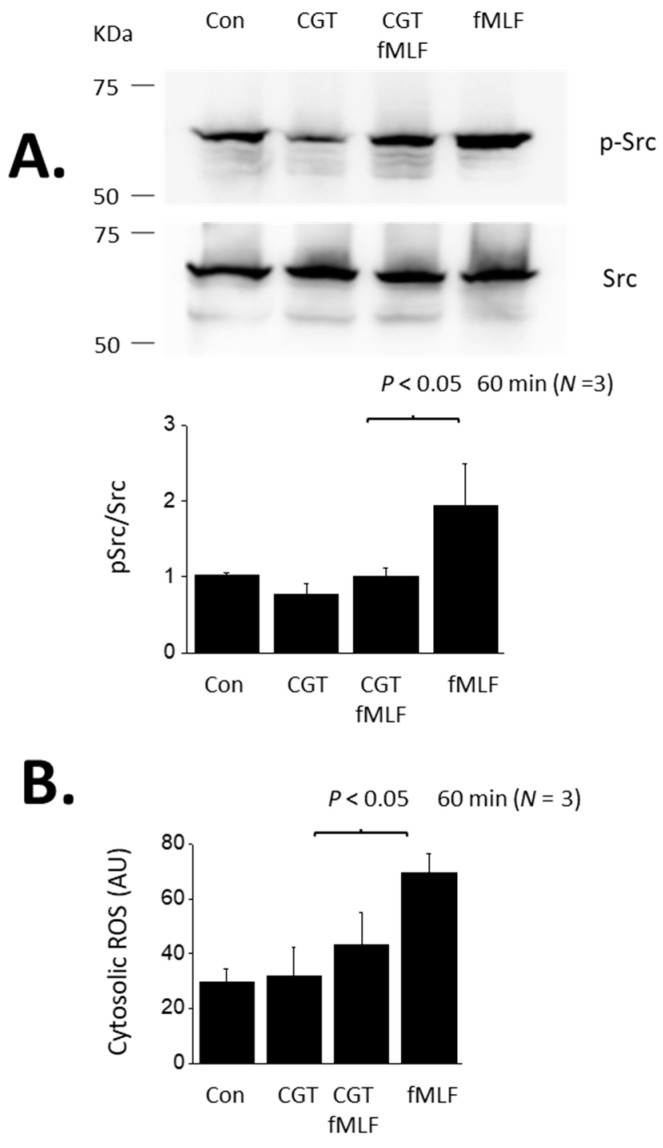
Src activation and cytosolic ROS accumulation evoked by a 60 min exposure to fMLF. (**A**) Immunoblot analyses showing an increase in Src phosphorylation evoked by a 60 min exposure to 1 µM fMLF and inhibition of this response by concomitant exposure to 1 µM CGT. (**B**) Increase in cytosolic ROS, in arbitrary fluorescence units (AU) evoked by 60-min exposure to fMLF and inhibition of the response by CGT.

Given the abundance of literature demonstrating a role for αVβ3-integrin in mediating transduction of suPAR signaling in podocytes, we carried out the converse experiment, which is to determine whether αVβ3-integrin is required for outside-in signaling evoked by fMLF in these cells. We observed that the αV-integrin inhibitor CGT (1 µM) caused a complete inhibition of Src activation evoked by a 24 h exposure to 1 µM fMLF (Figure 1C). Note that we have previously shown that CGT also blocks Src activation evoked by a 24 h exposure to 10 ng/mL suPAR [19,20,21]. In another set of experiments, we used a fluorometric assay to show that cytosolic ROS abundance is increased in podocytes following a 24 h exposure to either fMLF or suPAR (Figure 1D). Moreover, ROS production evoked by suPAR was blocked by WRW4 (suggesting a role for an FPR) whereas fMLF responses were completely blocked by CGT (suggesting a role for αVβ3-integrin) (Figure 1D). However, the actions of fMLF and suPAR differ in time course. We have previously shown that several hours of suPAR exposure is required for signal activation in podocytes [21]. Thus, Src activation and ROS accumulation cannot be detected with less than a 6 h exposure, and the vast majority of studies in this system have utilized 24 h exposures, including all of our previously published work. By contrast, a notable feature of GPCRs is that transduction through these systems is relatively fast, often in the range of seconds to minutes. In the present study we observed that fMLF-evoked signaling to Src and ROS occurs much more rapidly than signaling evoked by suPAR. Those responses are readily detected following a 60 min exposure to 1 µM fMLF (Figure 2). These relatively rapid responses to fMLF are also blocked by the integrin inhibitor CGT (Figure 2) as well as by WRW4 (data not shown). By contrast, suPAR is not able to cause activation of Src or to induce ROS accumulation within 60 min (see further below). 

**Figure 3:**
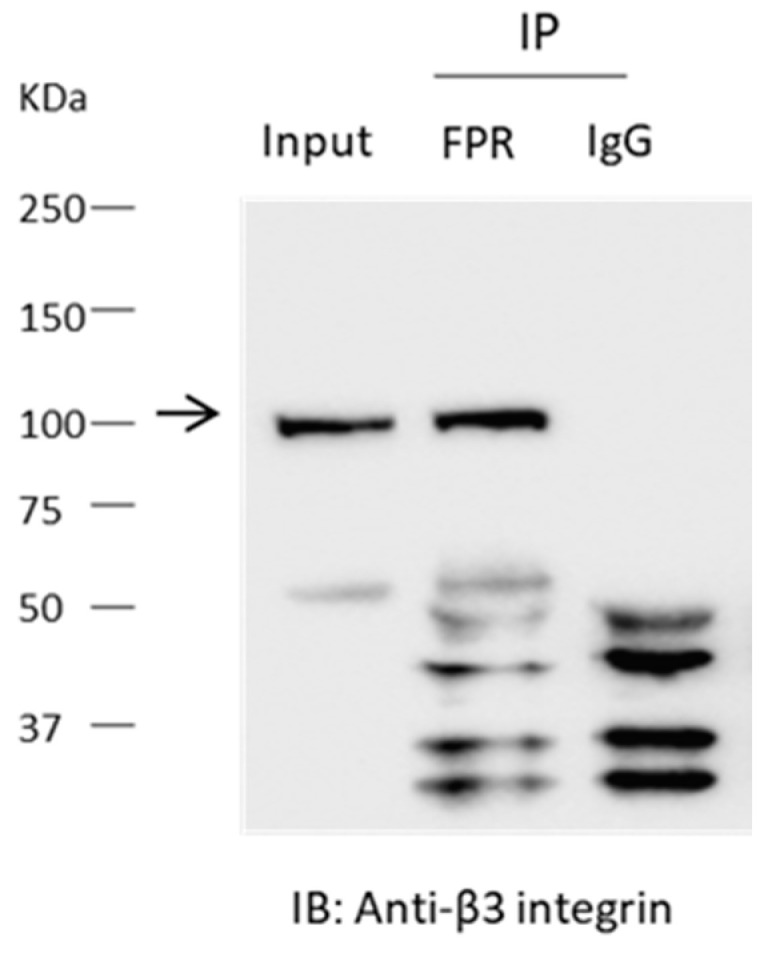
Interaction between FPR and β3-integrin detected by co-immunoprecipitation. Experiments were carried out on lysates of differentiated podocytes. The differentiated podocytes were not treated with agonists prior to preparation of the lysates. Immunoprecipitates obtained using an antibody against FPR1 or with IgG were analyzed by immunoblot using and antibody against β3-integrin. Note robust signal for β3 integrin in the precipitates prepared using the antibody targeting FPR1, but in in the lysate prepared with IgG. The molecular weight of the specific integrin signal is denoted by the arrow.

To explain these pharmacological observations, we hypothesized that αVβ3-integrin and an FPR are associated in a way that allows for cross-activation by the two receptor systems in mouse podocytes. That prediction was supported by an immunoprecipitation analysis. Thus, β3-integrin could be detected in precipitates prepared from podocyte lysates using an antibody against FPR1 (Figure 3), suggesting a close association between these signaling systems.

**Figure 4:**
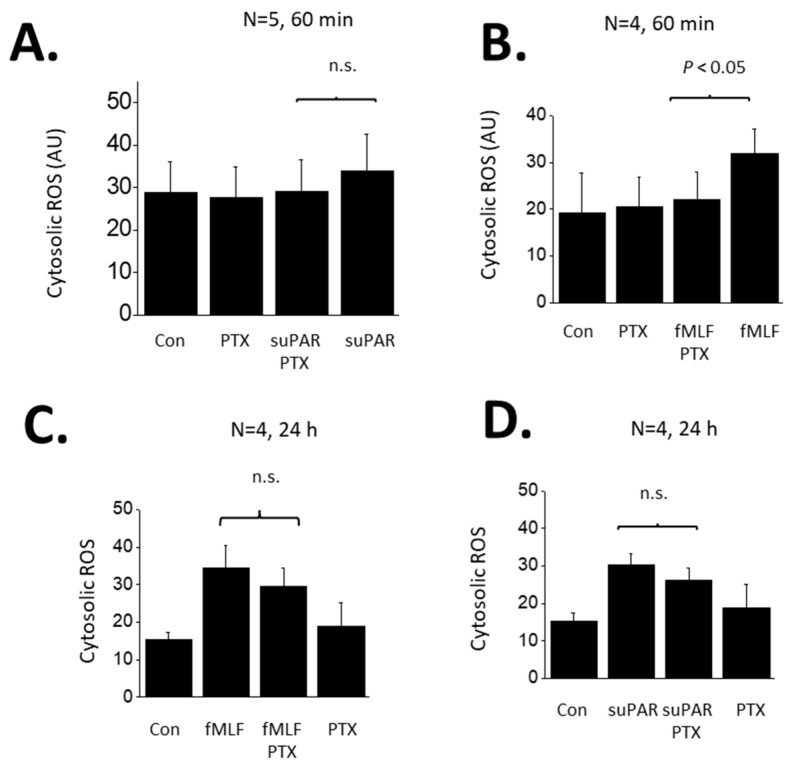
Role of G proteins in ROS generation evoked by suPAR and fMLF in podocytes. (**A**) Generation of cytosolic ROS evoked by 60 min exposure to 10 ng/mL suPAR. Note that this duration of suPAR exposure did not evoke a significant increase in cytosolic ROS that could be detected using the fluorescent probe. Pretreatment with PTX also had no effect. (**B**) A 60 min exposure to 1 μM fMLF evoked a significant increase in cytosolic ROS, and this effect was completely inhibited by pretreatment with PTX. (**C**) A 24 h exposure to 10 ng/mL suPAR evoked a significant increase in ROS that was also seen in cells pretreated with PTX. (**D**) A 24 h exposure to 1 μM fMLF evoked an increase in cytosolic ROS that was also observed in cells pretreated with PTX. In this and subsequent figures, n.s. denotes non-significant difference between groups underneath the brackets.

**Figure 5:**
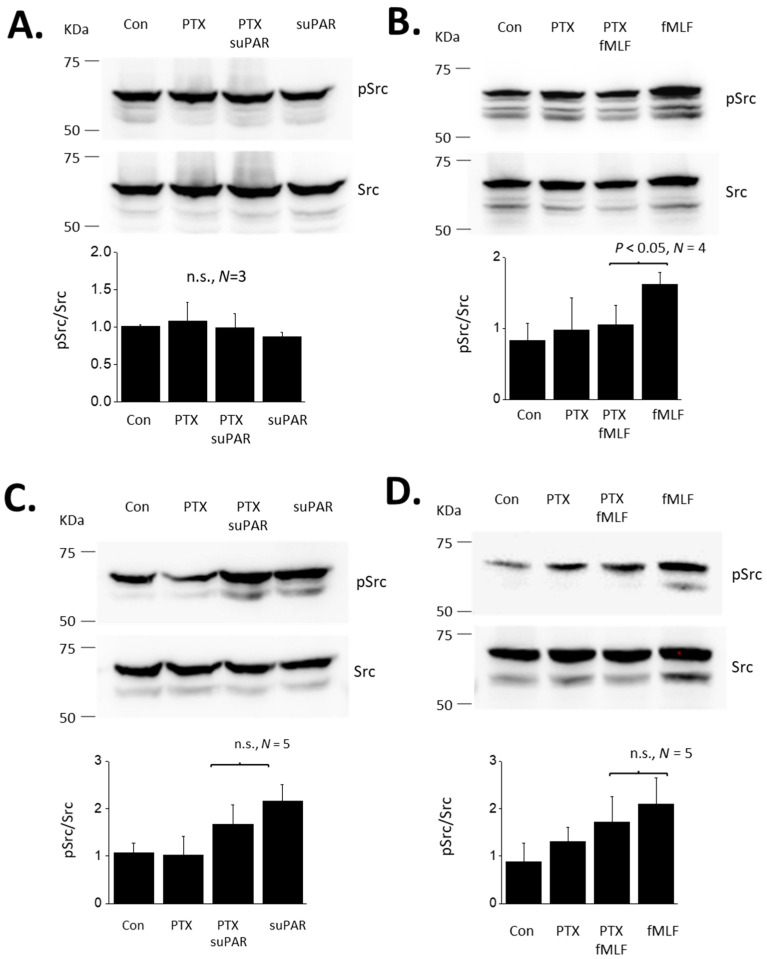
Role of G proteins in Src evoked by suPAR and fMLF in podocytes. (**A**) Src activation evoked by 60 min exposure to 10 ng/mL suPAR. Data are presented as in Figure 1. Note that this duration of suPAR exposure did not evoke Src activation. (**B**) A 60 min exposure to 1 μM fMLF evoked a significant increase in ROS activation, and this effect was completely inhibited by pretreatment with PTX. (**C**) A 24 h exposure to 10 ng/mL suPAR evoked a significant increase in Src activation that was also seen in cells pretreated with PTX. (**D**) A 24 h exposure to 1 μM fMLF induced Src activation that was also observed in cells pretreated with PTX.

FPRs are G-protein coupled receptors and are known to signal through pertussis toxin (PTX)-sensitive G proteins in many systems [34]. Therefore, we examined if the responses to suPAR and fMLF in podocytes are PTX-sensitive. In these experiments we examined responses to both 60 min and 24 h exposures to 1 µM fMLF or 10 ng/mL suPAR (Figure 4). As noted above, a 60 min exposure to suPAR was not sufficient to cause an increase in cytosolic ROS measured using a fluorometric assay (Figure 4A). By contrast, a 60 min exposure to fMLF evoked a significant increase in cytosolic ROS that was completely blocked by a 60 min pretreatment with 100 ng/mL PTX (Figure 4B). A 24 h exposure to either suPAR (Figure 4C) or fMLF (Figure 4D) evoked increases in cytosolic ROS. However, responses to these more sustained agonist exposures were mostly resistant to inhibition by PTX. The same pattern was obtained when Src phosphorylation was used as the readout, i.e., most or all of the signal remained in PTX-treated cells (Figure 5). Thus, the transduction pathway utilized by fMLF depends on the length of time that podocytes are exposed to this agonist, being PTX-sensitive at 60 min and mostly or completely PTX-resistant at 24 h. The 24 h suPAR responses measured here are mostly or completely PTX-resistant, and again we note that we do not detect responses to a 60 min exposure to suPAR using these readouts. 

**Figure 6:**
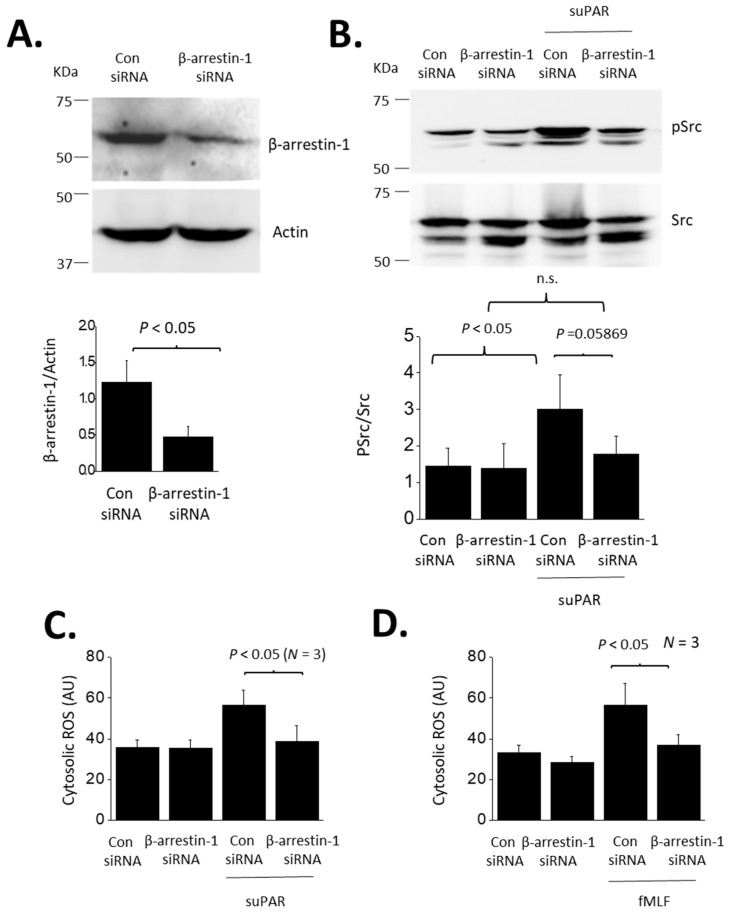
Role of β-arrestin-1 in suPAR signaling in podocytes. (**A**) Immunoblot showing significant reduction in β-arrestin-1 protein in cells transfected with a targeting siRNA, compared to cells transfected with a control siRNA. (**B**) β-arrestin-1 knockdown eliminated Src activation evoked by 24 h exposure to 10 ng/mL suPAR. The knockdown had no effect on untreated cells. (**C**) β-arrestin-1 eliminated cytosolic ROS accumulation evoked by 24 h exposure to suPAR. (**D**). β-arrestin-1 knockdown also eliminated ROS activation evoked by 24 h exposure to fMLF.

Inhibition of an agonist response by PTX normally implies transduction through a small subset of heterotrimeric G proteins that are activated by certain GPCRs. GPCRs can also signal through non-G protein pathways, for example by using β-arrestins to signal to various downstream components [37], including slit diaphragm proteins in the case of podocytes [38,39]. Therefore, we examined if the responses to suPAR are mediated by β-arrestins. In the present study we focused on BAR1, which we examined using siRNA methods. We confirmed that transient transfection with siRNA targeting BAR1 caused a marked reduction of BAR1 protein abundance as detected by immunoblot analysis (Figure 6A). Control cells were treated with a non-targeting siRNA.

**Figure 7:**
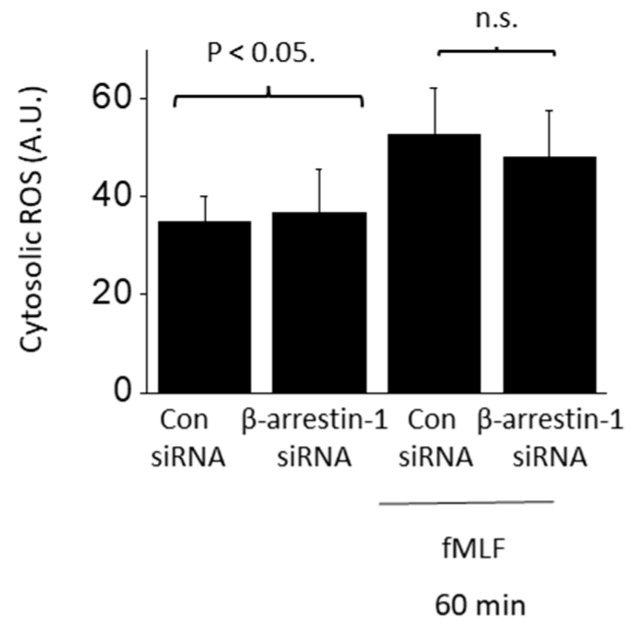
Rapid fMLF signaling does not require β-arrestin-1. Cytosolic ROS generation evoked by 60 min exposure to 1 µM fMLF persisted following siRNA knockdown of β-arrestin-1 (*N* = 3).

**Figure 8:**
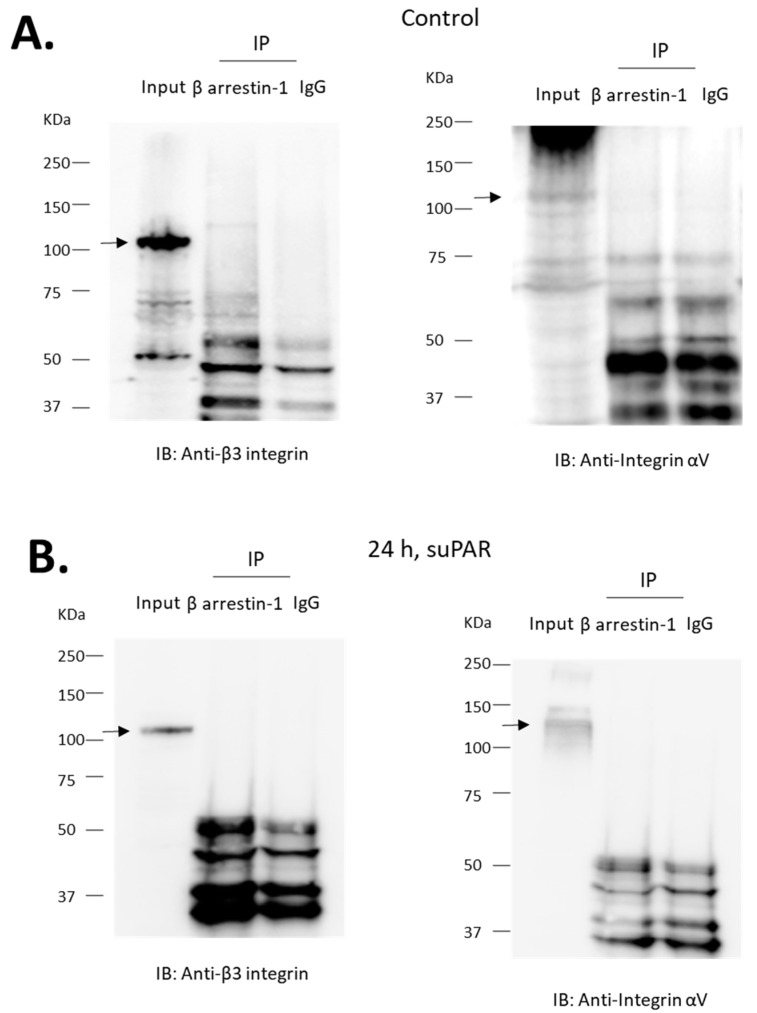
αV and β3-integrin subunits do not co-immunoprecipitate with β-arrestin-1. Experiments were carried out on lysates of differentiated podocytes. Immunoprecipitates obtained using an antibody against β-arrestin-1 or with IgG were analyzed by immunoblot using an antibody against αV or β3-integrin, as indicated. MW of specific signal for integrin is denoted by arrows. We were unable to detect the integrin subunits in precipitates prepared with the antibody against β-arrestin-1 or with IgG. This was observed in untreated cells (**A**) and in cells previously treated for 24 h with 10 ng/mL suPAR (**B**).

BAR1 knockdown abolished increases in both Src phosphorylation (Figure 6B) and cytosolic ROS (Figure 6C) evoked by 24 h exposure to 10 ng/mL suPAR. BAR1 knockdown also blocked increases in cytosolic ROS evoked by 24 h exposure 1 µM fMLF (Figure 6D). However, BAR1 knockdown did not result in significant inhibition of ROS accumulation evoked by a 60-min exposure to fMLF (Figure 7). Given that β-arrestins can interact with receptors other than GPCRs [30,31], we also addressed whether BAR1 interacts directly with αV- or β3-integrin subunits. We were unable to detect such an interaction by co-immunoprecipitation in either native podocytes or in podocytes treated for 24 h with 10 ng/mL suPAR (Figure 8).

## 4. Discussion

Increases in circulating suPAR have been implicated in a wide range of chronic clinical conditions, especially those that are associated with persistent and systemic inflammatory states, and that are common in the critical care environment [4,5]. In recent years extensive literature has emerged on the role of suPAR in both acute [10,12] and chronic [40] kidney disorders, and in processes whereby acute kidney injuries progress to CKD [41]. While it has long been known that suPAR is able to activate FPRs [22,23,24], most of the studies on suPAR in the kidney have focused on αVβ3-integrin as the primary cell surface receptor for suPAR [9,19]. We have recently shown that suPAR signaling in podocytes is more complex than a simple outside-in activation of αVβ3-integrin, as it also entails activation of RAGE, which in podocytes can be detected in a complex with αVβ3-integrin [21]. These results suggest that suPAR signal transduction in podocytes has complex features starting from its receptor on the cell surface, and they raise the possibility of numerous forms of receptor crosstalk and/or trans-activation. 

A key result in the present study is that FPR signaling is also essential for suPAR signal transduction in mouse podocytes. Thus, we observed that antagonists of FPRs (WRW4) and of αVβ3-integrin (CGT) can inhibit the actions of both suPAR and a prototypical FPR agonist (fMLF). It bears noting that there are eight different FPRs in mice, as compared to just three for humans [34]. We observed that endogenously expressed Fpr1 co-immunoprecipitates with the β3-integrin subunit. Mouse Fpr1 has about 77% sequence homology with human FPR1, and is considered the homolog of FPR1, but it also has some structural and functional features in common with human FPR2/ALX, including a relatively low affinity for fMLF [34,42]. 

FPRs typically activate PTX-sensitive G proteins in the Gαi family, and they undergo desensitization in response to more prolonged exposures to formyl peptide agonists in myeloid cells [34]. In previous studies we have examined responses to suPAR over time scales that are substantially longer than would typically be used in studies of GPCRs (24 h exposure) [19,20,21]. In the present study we examined the effects of the duration of suPAR or fMLF exposure on two different downstream readouts in podocytes. We observed that a 60 min exposure to fMLF caused activation of Src and increased accumulation of cytosolic ROS, whereas suPAR did not evoke either response within that time frame. However, podocytes continue to respond to fMLF even with 24 h of continuous exposure to this agonist. This is in marked contrast to what is observed in myeloid cells, which exhibit marked desensitization with continuous agonist exposure, the rate of which can depend on the nature of the readout studied, but which generally occurs in minutes [43]. As with other GPCRs, desensitization of FPRs occurs following phosphorylation of the receptors [34], and in some cases is associated with β-arrestin-dependent internalization of the receptors [44]. It is also notable that FPR desensitization can be relieved by activation of other types of receptors in the same cell [43]. 

Given the relatively complex pattern seen in podocytes, we examined the transduction processes in more detail, and observed that the responses to a 60 min exposure to fMLF were completely blocked by pretreatment with PTX, as would be predicted from numerous studies on myeloid cells [34]. We did not observe any response to suPAR following a 60 min exposure. However, Src activation and ROS accumulation evoked by a 24 h exposure to either fMLF or suPAR were readily detected even with concomitant exposure to PTX. Conversely, responses to a 60 min exposure to fMLF were robust following knockdown of BAR1, whereas BAR1 knockdown caused nearly complete inhibition of the responses to either fMLF or suPAR when those agonists were present for 24 h. 

**Figure 9:**
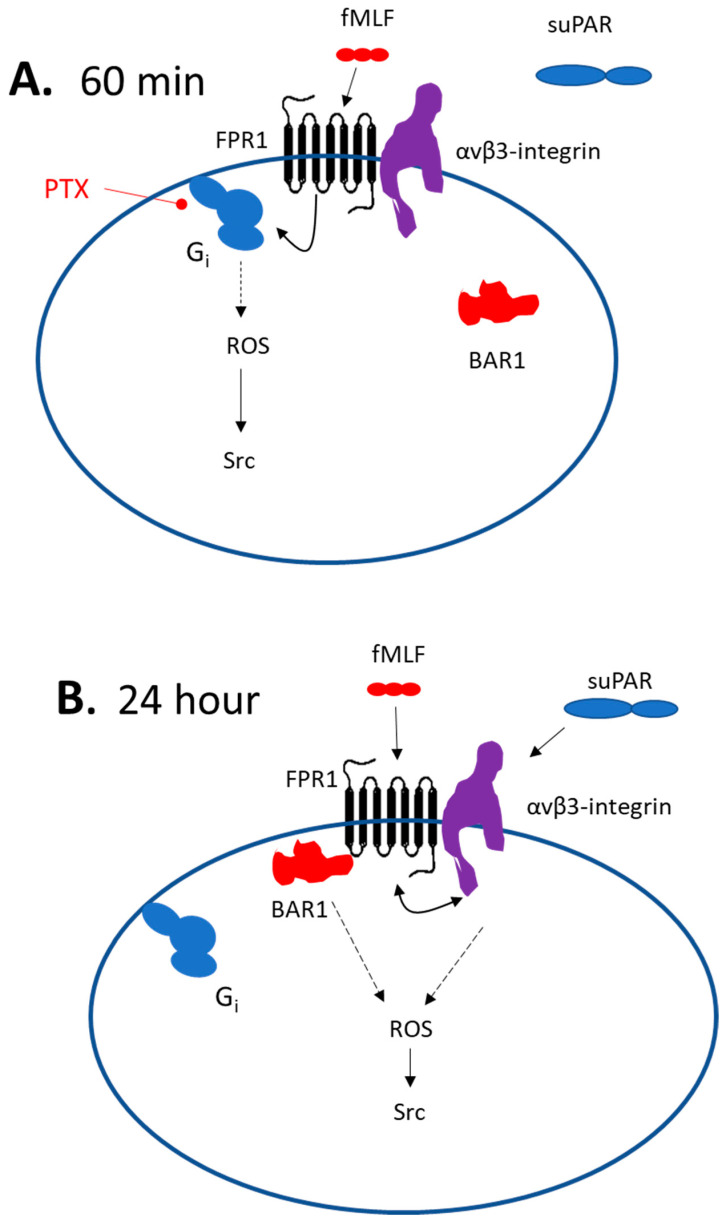
Models for suPAR and Fpr1 signaling in mouse podocytes. (**A**) Scheme describing 60 min exposure to fMLF or suPAR. In this situation the Fpr1 agonist signals to ROS and Src in a standard pathway that requires a PTX-sensitive Gαi. This response does not require BAR1. At this time point suPAR does not evoke detectable signals to ROS or Src, even though the β3-integrin subunit interacts with Fpr1. (**B**) With a 24 h exposure to either suPAR or fMLF, signals to ROS and Src require BAR1, which is presumably bound to Fpr1 at this time, thereby preventing interactions with Gαi. It is possible that Fpr1 causes BAR1-dependent trans-activation of αVβ3-integrin and vice versa. However, it is likely that other intermediary proteins would be required since we did not detect BAR1 interactions with either integrin subunit. This may entail some other type of GPCR, RAGE, or possibly other classes of receptors.

These data lend themselves to a variety of mechanistic interpretations, but one plausible model is shown in Figure 9. Short duration exposure to fMLF is proposed to cause activation of podocyte FPRs, presumably including Fpr1, which is transduced through a canonical pathway that entails PTX-sensitive Gαi, and from there to Nox2 and Src. As an aside, we note that Nox-2-dependent ROS generation is one of the most extensively studied responses to FPR activation in neutrophils [34]. In other words, this appears to be one of the canonical pathways. suPAR does not activate this pathway, possibly because it does not interact directly with podocyte FPRs, or because it is unable to induce a change in the conformation of the FPRs that would allow their interaction with Gαi-containing heterotrimeric G proteins. However, a 24 h exposure to either fMLF or suPAR evokes a BAR1-dependent and PTX-resistant pathway leading to ROS and Src. It is possible that interaction of BAR1 with podocyte Fpr1 allows it to trans-activate (and be trans-activated by) adjacent αVβ3-integrin receptors, which can enter a signaling conformation in response to stimuli originating on either side of the plasma membrane [45]. While the simplest mechanism would be if BAR1 acts as a simple scaffold to connect Fpr1 to αVβ3-integrin, we were unable to detect a direct biochemical interaction between BAR1 and either αV or β3 integrin subunits by co-immunoprecipitation in naïve podocytes or in podocytes that had been continuously exposed to suPAR for 24 h. It is possible that BAR1-integrin interactions in podocytes occur but are not stable enough to allow their detection by a co-immunoprecipitation method. However, it is also possible that some intermediary protein may be involved. One possibility is RAGE, which we have previously shown to be required for suPAR signal transduction in podocytes, and which interacts directly with αVβ3-integrin [21]. In fact, direct interactions between RAGE and FPRs have been reported in the transduction of signals evoked by amyloid beta (1–42) and S100 in glial cells [46]. Obviously, a 24 h period allows for many other hypotheses. 

A notable aspect of the present results, along with previous studies, is that three different structurally distinct classes of cell-surface receptors have now been implicated in suPAR signaling in podocytes: αVβ3-integrin [19,20], RAGE [21], and FPRs. All three types of receptors have numerous activating ligands and can function as pattern-recognition receptors (PRRs) that organize innate immune responses. Thus, conserved components of pathogenic microorganisms and viruses, and molecules released as a result of host tissue damage, collectively referred to as danger signals, bind to PRRs and thereby evoke signals that lead to T-cell activation [47]. For example, αVβ3-integrin is a binding domain for Herpes simplex virus [48] and the SARS-CoV-2 virus S-protein [49], and αVβ3-integrin interacts with Toll-like receptor 2 to coordinate the resulting secretion of cytokines [48]. RAGE is activated by a wide range of structurally distinct damage-associated and microbial pathogen-associated molecular pattern molecules, including bacterial endotoxins, viruses, alarmins, etc. [50]. The canonical agonists of FPRs are formylated peptides that are found in bacterial and mitochondrial proteins [25,26]. In this regard, it is worth noting that podocytes also express multiple Toll-like receptors [51,52].

These observations raise the question as to why podocytes express such a wide array of cell surface PRRs? Innate lymphoid cells that respond to danger signals via PRRs are highly enriched in barrier tissues, such as skin, GI tract, and airways, where they organize initial innate and adaptive immune responses [53]. By analogy to innate lymphoid cells, it may be useful to consider podocytes as a type of barrier cell. Certainly, from a topographical perspective, podocytes are continuous with the external environment via the renal tubules and lower urinary tract. By responding to a wide variety of extracellular ligands associated with infections, tissue damage, and innate immunity (including suPAR), they might allow for changes in glomerular barrier function. It has long been known that patients can present with transient proteinuria, for example during respiratory infections [54], sepsis [55], fevers [56] and following exercise [57]. These transient and relatively benign proteinurias are especially common in children [58,59]. A transient and reversible proteinuria can be triggered very quickly by danger signals and pathogen-derived molecules such as lipopolysaccharide [60]. It has been proposed that changes in glomerular filtration function that entail signaling to podocytes may represent an evolutionarily conserved strategy to facilitate renal clearance of pathogen-associated molecules [61,62]. However, it is possible that this response can become maladaptive if it is sustained, for example, due to dysfunctions of myeloid cells that lead to abnormally sustained secretion of certain immunomodulatory substances such as suPAR. Given the complexity of the cell surface receptor complexes that directly bind suPAR or danger signals, and the resulting potential for biased signaling, it is also possible that certain combinations of soluble factors would be more dangerous than others.

In summary, we have shown that FPRs play a role in suPAR signal transduction in mouse podocytes, and that these cells also respond to a formyl peptide FPR agonist. We have also shown that Fpr1 interacts with β3-integrin, that BAR1 is essential to evoke responses to suPAR, and also to sustained exposure to an FPR agonist. These observations suggest a variety of possible therapeutic targets for primary nephrotic syndromes, and possibly other conditions. 

## Data Availability

All data are available upon request.
